# Reduced uptake of mass treatment for schistosomiasis control in absence of food: beyond a randomized trial

**DOI:** 10.1186/s12879-015-1158-7

**Published:** 2015-10-14

**Authors:** Simon Muhumuza, Annette Olsen, Anne Katahoire, Fred Nuwaha

**Affiliations:** School of Medicine, Child Health and Development Center, Makerere University, Kampala, Uganda; Faculty of Health and Medical Sciences, Section for Parasitology and Aquatic Diseases, University of Copenhagen, Copenhagen, Denmark; School of Public Health, Makerere University, Kampala, Uganda

**Keywords:** Uptake of praziquantel, Feeding program, School children, Uganda

## Abstract

**Background:**

Sustaining high uptake of praziquantel is key for long-term control of schistosomiasis. During mass treatment in 2013, we randomized 12 primary schools into two groups; one group received education messages for schistosomiasis prevention for two months prior to mass treatment, while the other, in addition to the education messages, received a pre-treatment snack shortly before mass treatment. The uptake of praziquantel in the snack schools was 94 % compared to 79 % in the non-snack schools. During mass treatment in 2014, no snack was provided. We compared the uptake of praziquantel in 2014 to that in 2013 and attempt to explain the reasons for the observed differences.

**Methods:**

Serial cross sectional surveys were conducted among a random sample of children from the 12 primary schools, 1 month after mass treatment in 2013 and 2014 to measure uptake of praziquantel, reported side effects attributable to praziquantel and prevalence and intensity of schistosomiasis infection. Differences in the demographic and descriptive variables between the 2013 and 2014 samples were compared using chi squared tests for categorical variables and student’s *t*-test for geometric mean intensity of *S. mansoni* infection.

**Results:**

Uptake of praziquantel reduced from 93.9 to 78.0 % (*p* = 0.002) in the snack schools but was unchanged in the non-schools 78.7 and 70.4 % (*p* = 0.176). The occurence of side-effects attributable to praziquantel increased from 34.4 to 61.2 % (*p =* 0.001) in the snack schools but was unchanged in the non-snack schools; 46.9 and 53.2 % (*p* = 0.443). Although the prevalence of *S. mansoni* infection increased in both the snack and non-snack schools, the differences did not reach statistical significance;1.3 and 7.5 % (*p* = 0.051) and 14.1 and 22.0 % (*p* = 0.141), respectively. Similarly, the difference in the geometric mean intensity of *S. mansoni* infection in both the snack and non-snack schools was not statistically significant; 38.3 eggs per gram of stool (epg) and 145.7 epg (*p* = 0.197) and 78.4 epg and 322.5 epg (*p* = 0.120), respectively.

**Conclusion:**

Our results show that in absence of food, uptake of praziquantel reduced and the side-effects of the drug increased. However, the reduced uptake did not affect the prevalence and intensity of schistosomiasis among school children. Rescinding of the provision of the snack is what probably caused the reduction in uptake of treatment in the subsequent mass treatment cycle.

## Background

The goal of the Ugandan national schistosomiasis control program is morbidity control through regular mass treatment in endemic areas [1]. Annual school-based mass treatment with praziquantel is the cornerstone for schistosomiasis control among school-aged children. The school approach is based on the premises that it is cost-effective to deliver interventions through the school system because schools are widely distributed and that school teachers can assist to administer the drugs to the children [2–5]. The drawbacks of this strategy have been documented in many parts of the country. The reliance on volunteer teachers to distribute treatment, the fear of side-effects of praziquantel, low school attendance rates and the social, economic and political context in which mass treatment is rolled out profoundly affect the uptake of drugs among the targeted population [6–9]. In some locations, uptake of treatment has been found to be low [8–11]. The target is to treat at least 75 % of school-age children at risk of morbidity [12]. In a particular study conducted in Jinja district, less than 30 % of the school children took praziquantel during mass treatment in 2011. The fear of treatment, inadequate health education and lack of teacher support were some of the factors thought to influence the uptake [11]. In an effort to increase the uptake of treatment in 2012, the national control program motivated the teachers to distribute treatment through provision of incentives and intensified supervision with little success [13].

Praziquantel should be concurrently administered with food to mitigate the side effects of the drug and increase its uptake [14–16]. Studies assessing the use of food incentives have shown improved participation of children in health programs for treatment of schistosomiasis, HIV/AIDS and other diseases [16–19]. Furthermore, the use of food is associated with improved treatment outcomes and education benefits among school children [17, 20, 21]. In Uganda, the school feeding policy requires that parents take responsibility for feeding their children while at school [22]. However, due to the prevailing poverty in many parts of the country, most parents cannot afford to meet the cost of a daily meal for their children while at school [23]. In areas where annual treatment with praziquantel for schistosomiasis control is provided, most children take the treatment on an empty stomach and experience side effects [24, 25] and as such, reject the drug during the subsequent mass treatment programs [9, 26].

Strategies for improving uptake, such as provision of food to mitigate the side effects of praziquantel and motivate children to take treatment are required. Our study conducted in 2013 demonstrated the effectiveness of a pre-treatment snack on the uptake of praziquantel among school children [27]. This study compares the 2013 uptake with the 2014 uptake when no snack was provided. In addition, the reported side effects attributable to praziquantel and prevalence and intensity of schistosomiasis infection are compared.

## Methods

### Ethical considerations

Ethical permit to conduct the study was obtained from the School of Medicine Research and Ethics Committee, Makerere University and the Uganda National Council for Science and Technology. Clearance to conduct the study in the schools was obtained from the school management. Written informed consent from the parents and guardians of the children was obtained through half-day meetings held at the respective schools. Assent to participate in the study was obtained from all the children. Children identified with schistosomiasis and/or STH were treated with praziquantel and/or albendazole.

### Study design

Serial cross sectional surveys were carried out 1 month after mass treatment in 2013 and 2014. Both surveys employed similar quantitative methods of data collection.

### Study setting

The surveys were carried out in 12 primary schools of Walukuba division, Jinja district of south eastern Uganda, where intestinal schistosomiasis is highly endemic. This setting has been described in our earlier studies [11, 26, 27]. Briefly, annual school-based mass treatment is implemented as the main strategy for schistosomiasis control in the division. Treatment to the school children is distributed and registered by trained teachers who are supervised by sub-county health assistants and inspector of schools. However, uptake of praziquantel among school children in the area was low. Prior to the 2013 mass treatment, the 12 primary schools in the division were randomized into two groups; one received education messages for schistosomiasis prevention for two months prior to mass treatment, while the other, in addition to the education messages, received a snack before mass treatment. Each child in the snack schools received 500 ml of mango juice and a doughnut shortly before swallowing praziquantel during mass treatment. The education messages focused on the prevention aspects of schistosomiasis infection including taking preventive treatment (praziquantel) with food in order to avoid the side-effects of the drugs. Both the snack and the education messages were delivered by the teachers. These interventions were supported by the Danish Ministry of Foreign Affairs. The uptake of praziquantel in the snack schools (93.9 %) was considerably higher than in the non-snack schools (78.7 %). The occurrence of the side effects as well as the prevalence and geometric mean intensity of *S. mansoni* infection were considerably lower in the snack schools than in the non-snack schools [27]. Prior to the 2014 mass treatment, information about mass drug distribution was communicated through radio talk shows and spot messages. However, there was no snack provided to the children prior to treatment.

### Sample size

The uptake of praziquantel used to estimate the sample size for this study was derived from our previous study. It was assumed that uptake of treatment would decrease from 94 % in 2013 [27] to 75 % in 2014. At a 90 % power and a 95 % CI, the sample size required to test this difference was 85 in the snack schools and 85 in the non-snack schools according to STATA 12.0 (TX, USA). Because of the cluster design, a design effect of 6.3 from the previous study was applied to obtain a minimum sample size of 536 in the snack schools and 536 in the non-snack schools. In this study, 1,142 children were randomly selected from the 12 primary schools to participate in the 2014 study.

### Sampling and data collection

Similar sampling and data collection methods as those used in the previous study were employed [27]. Children in the two groups were randomly selected from grade (year) 4–6. A proportionate number of children selected from each school and grade was determined by probability proportional to size of the school and grade population. Systematic sampling was used to select the children from each grade to participate in the face to face interviews using a structured questionnaire and undergo a stool examination for *S. mansoni* infection using the modified Kato-Katz thick smear technique with a 41.7 mg template [28].

### Measures

Measures included self-reported uptake of praziquantel, occurrence of side-effects and prevalence and intensity of *S. mansoni* infection in the two groups. In addition, the socio-demographic characteristics of the children and their knowledge of schistosomiasis prevention were assessed. Uptake of praziquantel was measured on a score scale of 1–4. Children were asked whether they received treatment, and if they did, they were further asked the colour and the taste of the tablets, and whether they swallowed the drugs. For each positive response, a score of one was awarded. Children who reported to have received the drug, mentioned the true colour and taste of praziquantel and reported to have swallowed the tablets scored an aggregate of four and this was considered as true uptake of praziquantel. In addition, children who reported to have swallowed treatment were asked if they developed any side effects after swallowing the drug. Knowledge of schistosomiasis prevention was measured on a score scale of 1–3. Children were asked the different ways through which the infection can be transmitted and prevented. For each of the correct responses, a score of one was awarded. Children who mentioned at least one correct method of transmission and two correct methods of prevention scored an aggregate of three and were regarded as having correct knowledge of schistosomiasis prevention.

#### Laboratory diagnosis of *S. mansoni* infection

In order to determine the prevalence and intensity of *S. mansoni* infection, we examined one early morning stool specimen from each child on two consecutive days using the modified Kato-Katz faecal thick smear technique [28] with a 41.7 mg template. The Kato-Katz method was adopted because it is simple, low cost and uses an already established system to stratify infection intensities into different classes based on cut offs of egg-counts [29, 30]. Two slides per stool specimen were prepared and examined by two independent laboratory technicians. A discrepancy of more than 5 % in egg counts was validated by a third laboratory technician who read the results for confirmation and the counts harmonized. The egg counts were found to be over dispersed and thus were logarithmically transformed and intensities reported as geometric mean intensity (GMI) of eggs per gram of stool (epg) among positive cases only.

### Data management and statistical analysis

Data entry and validation was performed in Microsoft Access 2007 and exported to STATA 12.0 (TX, USA) for analysis. Analysis was done at both the cluster and individual levels. Mean and standard deviation (S.D) were used to describe continuous data. Differences in the demographic and descriptive variables between the 2013 and 2014 children samples were compared using chi squared tests for categorical variables. Student’s *t*-test was used to compare mean intensity of *S. mansoni* infection. Multivariable regression was done to identify the independent predictors of uptake of praziquantel. Clustering was adjusted for by dividing test statistics based on chi squared tests and the *t*- test with the design effect and the square root of the design effect [31, 32], respectively. The intracluster correlation coefficient (ICC) was 0.06 and the design effect was 6.3.

### Quality control

Data were collected by experienced research assistants who are fluent in the local language of Lusoga. Similar data collection tools as those used in 2013 were employed [27]. To ensure completeness and accuracy, data were checked before leaving the field and as such, no data were missing. The first author closely supervised the research assistants during data collection.

## Results

### Demographic characteristics

Overall, a total of 2,426 children in 12 primary schools were assessed one month after mass treatment in 2013 and 2014: 1,284 children in 2013 and another 1,142 in 2014. Of the 1,284 children assessed in 2013, 595 (46.3 %) were from the snack schools and 689 (53.7 %) were from the non-snack schools. Of the 1,142 children assessed in 2014, 496 (43.4 %) and 646 (56.6 %) were from the snack and non-snack schools, respectively. The mean age of children in the snack schools was 11.3 years (standard deviation [SD] 1.7) in 2013 and 11.6 years (SD 1.5) in 2014 while that in the non-snack schools was 11.7 years (SD 1.6) in 2013 and 12.2 years (SD 1.8) in 2014. Children in the snack and non-snack schools in the 2013 and 2014 samples were comparable in terms of age group, sex and distance from area of residence to the lake (*p* > 0.05) (Table 1).

**Table 1:** Comparison of the children in the two groups of schools after the 2013 and 2014 mass treatment

Characteristic	Non-snack schools				Snack schools			
	2013	2014	χ^2a^	*p*-Value	2013	2014	χ^2a^	*p*-Value
	*n* = (689) (%)	*n* = (646) (%)			*n* = (595) (%)	*n* = (496) (%)		
Age group								
07–11 years	291 (42.2)	301 (46.6)	0.379	0.538	310 (52.1)	241 (48.6)	0.190	0.663
12–16 years	398 (57.8)	345 (53.4)	------	------	285 (47.9)	255 (51.4)	------	------
Sex								
Female	343 (49.8)	345 (53.4)	0.256	0.613	296 (49.7)	270 (54.4)	0.349	0.555
Male	346 (50.2)	301 (46.6)	------	------	299 (50.3)	226 (45.6)	------	------
Distance from area of residence to Lake Victoria								
≤5 km	330 (47.9)	300 (46.4)	0.037	0.847	284 (47.7)	243 (49.0)	0.021	0.885
>5 km	359 (52.1)	346 (53.6)	------	------	311 (52.3)	253 (51.0)	------	------

^a^Adjusted for cluster design effect

### Self-reported uptake of praziquantel and occurrence of side-effects

A significant decrease in uptake of praziquantel from 93.9 % (95 % CI 91.7–95.7 %) in 2013 to 78.0 % (95 % CI 74.1–81.6 %) (*p* = 0.002) in 2014 was observed in the snack schools. However, uptake in the non-snack schools was unchanged; 78.7 % (95 % CI 75.4–81.7 %) in 2013 and 70.4 % (95 % CI 66.7–73.9 %) (*p* = 0.176) in 2014 (Table 2). The 2014 uptake in the snack and non-snack was comparable; 78.0 % (95 % CI 74.1–81.6 %) and 70.4 % (95 % CI 66.7–73.9 %) (*p* = 0.250), respectively.

**Table 2:** Characteristics of the snack and non-snack school after the 2013 and 2014 mass treatment

Characteristic	Non-snack schools				Snack schools			
	2013	2014	Test Statistic^a^	*p*-Value	2013	2014	Test Statistic^a^	*p*-Value
	*n* = (689) (%)	*n* = (646) (%)			*n* = (595) (%)	*n* = (496) (%)		
Knowledge of schistosomiasis prevention (%)								
Yes	595 (86.4)	362 (56.0)	χ^2^ = 23.732	<0.001	500 (84.0)	308 (62.1)	χ^2^ = 10.574	0.001
No	94 (13.6)	284 (44.0)	------	------	95 (16.0)	188 (37.9)	------	------
Eaten food prior to mass treatment (%)								
Yes	270 (49.8)	148 (32.5)	χ^2^ = 4.708	0.030	519 (92.8)	211 (54.4)	χ^2^ = 30.103	<0.001
No	272 (50.2)	307 (67.5)	------	------	40 (7.2)	177 (45.6)	------	------
Self-reported uptake of praziquantel (%)								
Yes	542 (78.7)	455 (70.4)	χ^2^ = 1.829	0.176	559 (93.9)	387 (78.0)	χ^2^ = 9.231	0.002
No	147 (21.3)	191 (29.6)	------	------	36 (6.1)	109 (22.0)	------	------
Side-effects attributable to praziquantel (%)								
Yes	254 (46.9)	242 (53.2)	χ^2^ = 0.589	0.443	192 (34.3)	240 (61.2)	χ^2^ = 10.486	0.001
No	288 (53.1)	213 (46.8)	------	------	367 (65.7)	152 (38.8)	------	------
Reported side-effects attributable to praziquantel (%)								
Abdominal pain	133 (52.4)	180 (74.4)	χ^2^ = 6.195	0.013	109 (56.8)	157 (65.4)	χ^2^ = 3.202	0.074
Dizziness	61 (24.0)	27 (11.2)	------	------	45 (23.4)	40 (16.7)	------	------
Vomiting	19 (7.5)	11 (4.6)	------	------	7 (3.6)	25 (10.4)	------	------
Diarrhoea	31 (12.2)	7 (2.9)	------	------	27 (14.1)	12 (5.0)	------	------
Headache)	10 (3.9)	17 (7.0)	------	------	4 (2.1)	6 (2.5)	------	------
*S. mansoni* infection status (%)								
Positive	97 (14.1)	142 (22.0)	χ^2^ = 2.165	0.141	8 (1.3)	37 (7.5)	χ^2^ = 3.819	0.051
Negative	592 (85.9)	504 (78.0)	------	------	587 (98.7)	459 (92.5)	------	------
Intensity of *S. mansoni* infection (epg)								
GMI epg	78.4	322.5	*t* =2.412	0.120	38.3	145.7	*t* =1.662	0.197

^a^Adjusted for cluster design effect

A considerable increase in the occurrence of side-effects attributable to praziquantel was observed in the snack schools in 2013; from 34.4 % (95 % CI 31.5–39.8 %) in 2013 to 61.2 % (95 % CI 56.2–66.1 %) (*p =* 0.001) in 2014. However, the occurrence of side-effects was unchanged in the non-schools; 46.9 % (95 % CI 42.2–50.7 %) in 2013 and 53.2 % (95 % CI 48.5–57.9 %) (*p* = 0.443) in 2014. A significant decrease in the proportion of children who reported to have eaten something prior to mass treatment was noted in both the snack and non-snack schools from 92.8 % (95 % CI 90.4–98.4) to 54.4 % (95 % CI 48.8–58.9 %) (*p* < 0.001) in 2014 and from 49.8 % (95 % CI 45.5–54.1 %) to 32.5 % (95 % CI 28.2–37.0 %) (*p* < 0.001) in 2013, respectively. The frequency of side-effects in the snack and non-snack schools in 2014 was comparable; 61.2 % (95 % CI 56.2–66.1 %) and 53.2 % (95 % CI 48.5–57.9 %) (*p* = 0.349), respectively. The proportion of children who reported to have eaten something prior to mass treatment in 2014 was 54.4 % (95 % CI 48.8–58.9 %) in the snack schools compared to 32.5 % (95 % CI 28.2–37.0 %) (*p* = 0.011) in the non-snack schools.

### Prevalence and mean intensity of *S. mansoni* infection

Prevalence of *S. mansoni* infection in the snack schools was unchanged;1.3 % (95 % CI 0.6–2.6 %) in 2013 and 7.5 % (95 % CI 5.3–10.1 %) (*p* = 0.051) in 2014. Similarly, the prevalence of *S. mansoni* infection in the non-snack schools was unchanged; 14.1 % (95 % CI 11.6–16.9 %) in 2013 and 22.0 % (95 % CI 18.8–25.4 %) (*p* = 0.141) in 2014. A statistically significant difference in the prevalence of *S. mansoni* infection was noted between the snack and non-snack schools in 2014; 22.0 % (95 % CI 18.8–25.4 %) and 7.5 % (95 % CI 5.3–10.1 %) (*p* = 0.008), respectively.

Although the mean intensity of *S. mansoni* infection increased in both the snack and non-snack schools, the differences did not reach statistical significance; 38.3 epg (95 % CI 21.8–67.2) in 2013 and 145.7 epg (95 % CI 92.6–229.5) (*p* = 0.197) in 2014 and 78.4 epg (95 % CI 60.6–101.5) in 2013 and 322.5 epg (95 % CI 258.1–403.1) (*p* = 0.120) in 2014, respectively (Table 2).

### Knowledge of schistosomiasis prevention

The proportion of children with correct knowledge of schistosomiasis prevention in both the snack and non-snack schools significantly reduced from 84.0 % (95 % CI 80.5–87.1 %) in 2013 to 62.1 % (95 % CI 56.3–67.5 %) (*p* < 0.001) in 2014 and from 86.4 % (95 % CI 83.4–89.0 %) in 2013 to 56.0 % (95 % CI 50.8–61.3 %) (*p* < 0.001) in 2014, respectively. However, knowledge of schistosomiasis prevention in the snack and non-snack schools in 2014 was comparable; 62.1 % (95 % CI 57.7–66.4 %) and 56.0 % (95 % CI 52.1–59.9 %) (*p* = 0.412), respectively.

### Predictors of uptake of praziquantel

Through a step wise backward elimination method, variables with *p* < 0.05 from the unadjusted model (distance from school to the lake, sensitized about schistosomiasis, knowledge of schistosomiaisis prevention and eaten food prior to mass treatment) were considered for inclusion into the multivariable adjusted model. The only variable retained in the final model was eating food prior to mass treatment (adjusted risk ratio (ARR) 1.01, 95 % CI 1.01–1.02, *p* = 0.020) (Table 3).

**Table 3:** Estimated crude risk ratios (CRR) and adjusted risk ratio (ARR) and their 95 % CI from the final logistic regression mixed-effects model for treatment uptake

Variable	CRR (95 % CI)	*p*-Value	ARR (95 % CI)	*p*-Value
Distance from school to the lake				
≤5 km	1.13 (1.03–1.24)	0.009	1.00 (0.99–1.00)	0.343
Sensitized about schistosomiasis prevention				
Yes	1.38 (1.18–1.61)	<0.001	1.01 (0.01–1.03)	0.247
Correct knowledge of schistosomiasis prevention				
Yes	1.24 (1.13–1.36)	<0.001	1.02 (0.99–1.04)	0.174
Eaten food prior to mass treatment				
	1.01 (1.00–1.02)	0.020	1.01 (1.00–1.02)	0.020

Adjusted for age and sex

## Discussion

This study found that the 2014 uptake of praziquantel was lower than the 2013 uptake although the prevalence and intensity of *S. mansoni* infection was unchanged between the years. The reported frequency of side-effects in 2014 was higher than that in 2013. Knowledge of schistosomiasis prevention was lower in 2014 than in 2013.

The observed decrease in uptake of treatment in 2014 may be attributable to lack of food for mitigating the side-effects of praziquantel. During the 2013 mass treatment, food to alleviate the side-effects and motivate the children to take treatment was provided to children with high levels of success [27]. The fear of side-effects as a major reason for non-uptake of praziquantel has been previously reported [8, 11, 13]. Food high in carbohydrate content increases the absorption of praziquantel form the gastro-intestinal tract, enhances its bioavailability and lowers the odds of side-effects [14–16]. During the 2014 mass treatment, only 54.4 % compared to 92.8 % of the children in the snack schools in 2013 reported to have taken food prior to mass treatment, the majority reporting maize porridge as the major type of food taken. The other types of food reported included pancakes, maize cobs and dry tea. This implies that a large proportion of children in 2014 took treatment on an empty stomach with the risk of widespread side-effects. The drug distribution strategies in the non-snack schools in 2013 and 2014 were similar, with no specific measures to mitigate the side-effects and motivate children to take treatment on both occasions. This probably explains the unvarying uptake levels and occurrence of side-effects among children in this group. These data suggest that the reasons for the low uptake identified in our previous studies [11, 13, 26] such as, inadequate information about schistosomiasis prevention, insufficient preparation and facilitation of teachers to distribute treatment and the fear of side-effects of praziquantel, especially when the drug is taken on an empty stomach are probably accurate and the measures suggested, such as provision of food in combination with education messages prior to treatment [27] are necessary to maintain high treatment coverage. A detailed cost-effectiveness analysis of provision of indigenous, locally available and relatively low cost food, such as maize porridge, during mass treatment should be undertaken and if found cost-effective, provision of food should be integrated into school-based mass treatment. A multi-pronged strategy for provision of food by the national control program, parents or the Government could be used.

The prevalence and intensity of *S. mansoni* infection after mass treatment in 2013 and 2014 were unchanged in both groups of schools. Although the realized uptake in both the non-snack (70.4 %) and the snack schools (78.0 %) during the 2014 mass treatment was lower than the 2013 uptake (78.7 and 93.9 %), the 2014 uptake was adequate to maintain the levels of infection but inadequate to significantly reduce the prevalence rates and intensity of the infection. In high transmission areas, chemotherapy with praziquantel is effective in reducing morbidity but the prevalence and intensity of infection return to pre-treatment levels within a short time if no other means of control are applied, even when treatment coverage is high [33]. In the study area, the majority of the children frequently visit the lake to fetch water, bathe, wash, fish and to swim [10, 11] and are thus at high risk of infection and re-infection. Sustained drop in infection can be achieved through a combination of interventions including preventive chemotherapy, health education, cessation of poor human sanitation practices and improved access to safe water [34, 35]. Each of these strategies is important but not sufficient when applied in isolation. Inadequate resources and capacity for such measures are major obstacles to sustainable schistosomiasis control in developing countries [36]. In the study area, school-based mass treatment with praziquantel is the mainstay for schistosomiasis control among school-age children. The challenges associated with this strategy have been discussed elsewhere [6, 7, 37, 38].

Knowledge of schistosomiasis prevention in both groups of schools in 2014 was considerably lower than that in 2013, a manifestation of the insufficient health education provided in 2014. Prior to the 2013 mass treatment, education messages tailored to the knowledge gap in schistosomiasis prevention were provided to all children in both groups of schools with high levels of knowledge registered in the schools. In 2014, less than a half of the children in both the snack and non-snack schools reported to have been sensitized about schistosomiasis prior to mass treatment. As such, a significant reduction in the proportion of children with adequate knowledge of schistosomiasis prevention was noted in both groups of schools. Health education and other behavioural change interventions are effective strategies for increasing compliance to treatment for schistosomiasis [39–41]. In the absence of these interventions, there is a risk that the targeted populations may resist treatment [9, 39].

## Study limitations

Children who participated in both studies were selected at random. It is possible that some of the children examined in 2013 could have been re-examined in 2014 and provided similar responses on both occasions. However, the correlation between self-reported uptake and *S. mansoni* infection and intensity validated the self-reported uptake as accurate. A negative linear relationship between uptake of praziquantel and prevalence as well as intensity of *S. mansoni* infection was observed: In schools with high uptake, the prevalence and intensity of *S. mansoni* infection was much lower than in schools with low uptake. Secondly, the Kato-Katz technique adopted for this study analyses a small amount of stool (41.7 mg) and therefore has a relatively low capacity to detect eggs from specimens of low intensity infections [30, 42]. The sensitivity of the method was increased by examining two stool samples from each child and preparing two thick stool smears from each sample.

## Conclusions

This study shows that in the absence of food to motivate children to take treatment and to mitigate the side-effects of praziquantel, uptake of praziquantel reduces and the side-effects of the drug increase. Thus, strategies for maintaining high treatment coverage are needed. Such strategies should include continued sensitization and education of the children and provision of food at school to mitigate the side-effects attributable to praziquantel treatment and motivate children to take treatment.
